# Mapping brain function during naturalistic viewing using high-density diffuse optical tomography

**DOI:** 10.1038/s41598-019-45555-8

**Published:** 2019-07-31

**Authors:** Andrew K. Fishell, Tracy M. Burns-Yocum, Karla M. Bergonzi, Adam T. Eggebrecht, Joseph P. Culver

**Affiliations:** 1 Washington University School of Medicine, Division of Biology and Biomedical Sciences, St. Louis, USA; 2 Washington University School of Medicine, Mallinckrodt Institute of Radiology, St. Louis, USA; 3 Indiana University, Department of Psychological and Brain Sciences, Bloomington, USA; 4 University of Pennsylvania, Department of Anesthesia and Critical Care, Philadelphia, USA; 5 University of Pennsylvania, Department of Physics, Philadelphia, USA; 6 Washington University, Department of Physics, St. Louis, USA; 7 Washington University, Department of Biomedical Engineering, St. Louis, USA

**Keywords:** Biophotonics, Perception, Sensory processing

## Abstract

Naturalistic stimuli, such as movies, more closely recapitulate “real life” sensory processing and behavioral demands relative to paradigms that rely on highly distilled and repetitive stimulus presentations. The rich complexity inherent in naturalistic stimuli demands an imaging system capable of measuring spatially distributed brain responses, and analysis tools optimized for unmixing responses to concurrently presented features. In this work, the combination of passive movie viewing with high-density diffuse optical tomography (HD-DOT) is developed as a platform for naturalistic brain mapping. We imaged healthy young adults during free viewing of a feature film using HD-DOT and observed reproducible, synchronized cortical responses across a majority of the field-of-view, most prominently in hierarchical cortical areas related to visual and auditory processing, both within and between individuals. In order to more precisely interpret broad patterns of cortical synchronization, we extracted visual and auditory features from the movie stimulus and mapped the cortical responses to the features. The results demonstrate the sensitivity of HD-DOT to evoked responses during naturalistic viewing, and that feature-based decomposition strategies enable functional mapping of naturalistic stimulus processing, including human-generated speech.

## Introduction

Optical neuroimaging techniques enable functional brain imaging in naturalistic settings unavailable to imaging modalities with highly constrained imaging environments such as functional magnetic resonance imaging (fMRI)^1,2^. For instance, functional near-infrared spectroscopy (fNIRS) enables functional brain imaging of social interactions or unconstrained movements^3–9^. Naturalistic imaging paradigms more closely recapitulate real-life conditions than experiments relying on tightly controlled stimuli, such as assessing speech perception with single sentence presentations, or mapping retinotopic organization of visual cortex using flashing checkerboard patterns^10–14^. Further, naturalistic paradigms are highly engaging, contain multi-modal content, and may be particularly well suited for populations (e.g. young children) unable make overt behavioral responses or perform a repetitive or predictable task^15–18^. In addition to social interactions and natural movements, naturalistic imaging paradigms have included free viewing of movies and television shows^19–21^. Naturalistic viewing paradigms employing movies or television shows enable repeatability and control over stimulus presentation, like experiments incorporating simplified and distilled stimuli, but preserve the richness and greater ecological validity associated with more unconstrained naturalistic paradigms.

Naturalistic viewing tasks have been extensively studied using other brain imaging modalities, including fMRI^20^, EEG^22^ and MEG^23^. Work using fMRI has established both practical and neuroscientific advantages of naturalistic viewing experiments. From a practical perspective, participants passively viewing a movie during brain imaging, particularly children, tend to move less relative to other passive tasks, such as resting-state paradigms, thereby reducing the pernicious effects of image artifacts related to head motion^24–26^. In the cognitive neuroscience literature, naturalistic viewing tasks have been shown to reliably provide synchronized cortical responses across participants, show sensitivity to subsequent memory of the movie content, and modulate across typical and atypical developmental trajectories^20,27–31^. Further, comprehension of the narrative elements of the stimulus is not constrained to a single sensory modality, further emphasizing the richness contained within naturalistic stimuli such as movies^32^. Though some optical studies have utilized naturalistic settings such as real-life interactions, the methodological and scientific appeal of repeatable and tunable narrative movie viewing paradigms, in general, have yet to be fully leveraged using optical neuroimaging^3^.

Naturalistic viewing simultaneously and reliably engages multiple cortical processing systems, including those related to processing the movie’s auditory/visual content and narrative structure^19,20^. These systems are spatially distributed across the cortex, underscoring the need for a large field-of-view to capture the multi-modality responses. Furthermore, the complexity of information contained within the stimulus demands high spatial resolution, in order to map features within a modality (e.g. visual categories) to cortical structures related to processing those features. As with other whole-brain paradigms, such as resting state functional connectivity, imaging systems with higher space bandwidth product (~FOV/Resolution) provide more powerful readouts of movie-evoked responses. Therefore, in comparison to traditional sparse fNIRS systems, optical neuroimaging techniques such as high-density diffuse optical tomography (HD-DOT), which utilize a densely arranged array of measurements across a broad field-of-view, are better suited for mapping movie-evoked responses^33–35^.

The central goal of the present work is to evaluate the functional mapping performance of naturalistic movie viewing combined with a large field-of-view HD-DOT system in healthy young adults. Cortical synchronization, as indexed by the correlation coefficient between the brain responses to repeated movie viewings, has been demonstrated using other imaging modalities including EEG^22^, ECoG^36^, MEG^23^, and fMRI^20,37,38^. Cortical maps of the correlation strength between runs during naturalistic viewing highlight the broad constellation of regions reliably involved in stimulus processing. Further, if HD-DOT is sensitive to complex, multi-modal cortical responses associated with naturalistic viewing, we hypothesize that highly reproducible, synchronized, cortical responses will be measurable across regions related to both sensory (auditory/visual) and higher-order cognitive (e.g. linguistic) processing.

A limitation of spatially mapping the correlation coefficient between brain responses measured across repeated viewings is that this style of analysis is agnostic to specific components of the stimulus, such as speech or visual motion, that are relevant to mapping cortical information processing. In contrast, feature extraction tools provide a powerful technique for parameterizing individual movie features and subsequently identifying regions related to processing those features during naturalistic viewing^19,39,40^. Accordingly, the second analysis developed in this paper is an approach that maps feature-specific cortical responses during naturalistic viewing. Like cortical responses mapped with reductive, non-naturalistic stimuli, these feature maps relate measured brain responses to task-related information processing demands.

## Methods

### Participants

Participants in this experiment were healthy young adults, recruited from the Washington University community. All participants gave written informed consent to participate in the experiment, which was approved by and carried out in accordance to the Human Research Protection Office at Washington University School of Medicine. Participants, all right-handed native English speakers, self-reported no history of neurological or psychiatric illness. In total, 12 participants were enrolled in the naturalistic viewing experiment (aged 23.5–29.4 years; 6 female). Of the 12 initial participants, 10 are included in the analyses reported below, as two participants were excluded due to falling asleep during one of the two imaging sessions.

### Stimuli and experimental procedure

Participants underwent an HD-DOT cap fit procedure lasting approximately 5–10 minutes, guided by real-time readouts of measurement light level, signal-to-noise, and optode-scalp coupling coefficients. Following cap fit, participants began the naturalistic viewing experiment. Informed by previous fMRI studies^20^, all participants in this experiment viewed a 30-minute segment from the feature film, *The Good*, *the Bad*, *and the Ugly*, directed by Sergio Leone^41^. As published previously using this stimulus, participants viewed minutes 16:48 to 46:48^20^. During an imaging session, participants viewed the same 30-minute segment two times, and each participant completed at least two imaging sessions on separate days. During passive movie viewing, unless otherwise specified, participants were instructed to relax, remain still, and watch the movie as they would normally, outside of the laboratory. The stimulus was presented on a 20-inch (diagonal) liquid-crystal display with 1080 × 760 pixels, positioned 75 cm from the participant’s nasion, subtending a vertical view angle of 23 degrees, and a horizontal view angle of 30 degrees. The stimulus was presented using the Psychophysics Toolbox 3 package for MATLAB (2010b)^42^.

### HD-DOT Instrumentation

**Figure 1:**
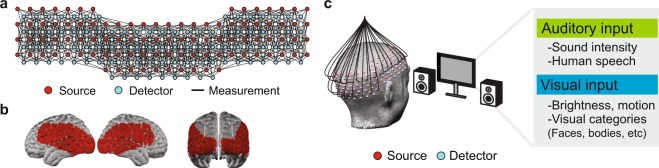
HD-DOT Instrumentation. (**a**) The HD-DOT instrument used in the naturalistic viewing experiments consisted of a 96 source, 92 detector array, resulting in a dense grid of measurements used to produce spatially-resolved maps of brain hemodynamics. Black lines indicate measurements used in image reconstruction from a representative participant. (**b**) The cortical field-of-view resulting from the optode arrangement. (**c**) During naturalistic viewing, participants watched a clip from a feature film, while undergoing multi-modal sensory stimulation resulting from a hierarchical set of visual and auditory features.

The large field-of-view HD-DOT instrument used in this experiment (Fig. 1) has been described in detail in prior work using this instrument^35^. In brief, this custom-built continuous wave instrument consists of 96 LED sources illuminating the head at two wavelengths (750 nm and 850 nm), and 92 avalanche photo diode detectors (Hamamatsu C5460-01), coupled to the head using 4.2 m long fiber-optic bundles (CeramOptec, 2.5-mm diameter bundles of 50 µm fibers). The weight of the 188 fibers was managed using an extruded aluminum frame and series of collinear rings surrounding the participant, ensuring that participants do not bear any of the fiber weight.

Fibers were affixed to the scalp using a custom-built imaging cap, which positions optodes such that first-through fourth-nearest neighbor separations are 1.3, 3.0, 3.9, and 4.7 cm, respectively. Using previously published temporal, frequency, and spatial encoding patterns, the HD-DOT system achieves an overall framerate of 10 Hz^35^. In a typical participant, this system configuration yielded over 1,200 source-detector measurements (per wavelength), which were then converted into voxelated movies of brain hemodynamics as specified below.

### HD-DOT Image Reconstruction

Image reconstruction occurs in five separate phases: light-level measurement pre-processing, anatomical light modeling, image reconstruction, spectroscopy and spatial normalization. The measurement pre-processing and image reconstruction steps followed previously published procedures using the same HD-DOT instrument^35^. Raw detector light levels were first converted to time-series log-ratio data. Log-ratio data was generated by taking the logarithm of the ratio of the instantaneous light level and the source-detector measurement’s mean value across the entire run. In this approach, the baseline is therefore defined as the measurement’s mean value across the entire run. Next, any measurements with a temporal variance exceeding 7.5% were considered to be contaminated by non-physiological variance (e.g. head motion) and excluded from image reconstruction for the entire run. The percentage of measurements retained for each source-detector separation in this sample was (Mean ± SD): 99.7 ± 1% of first nearest-neighbors, 97.0 ± 3% of second nearest-neighbors, 83.6 ± 11% of third-nearest neighbors, and 40.0 ± 9% of fourth nearest-neighbors. Consequently, the exact set of measurements used in image reconstruction varied on a run-by-run basis (Supplemental Fig. 1). However, the measurement density afforded by the HD-DOT instrument ensures that a given voxel is over-sampled by multiple measurements and minimizes any potential sampling dropout caused by the removal of any single measurement. The measurements that passed the variance threshold were then high-pass filtered (*f* > 0.02 Hz) to remove long term drift. Next, systemic and superficial signals, which were approximated by averaging all first nearest-neighbor measurements, were regressed out of all measurements. Measurements were then low-pass filtered (*f* < 0.5 Hz).

For anatomical light modeling, the non-linear ICBM152 atlas from the Montreal Neurological Institute was used to generate a wavelength-dependent forward model of light propagation through five non-uniform tissue compartments with tissue specific optical properties (units mm^−1^): scalp (*μ*_*a*,750_ = 0.017; *μ*_*a*,850_ = 0.019; *μ*_*s*,750_′ = 0.74; *μ*_*s*_,_850_′ = 0.64), skull (*μ*_*a*,750_ = 0.012; *μ*_*a*,850_ = 0.014; *μ*_*s*,750_′ = 0.94; *μ*_*s*,850_′ = 0.84), grey matter (*μ*_*a*,*750*_ = 0.018; *μ*_*a*,*850*_ = 0.019; *μ*_*s*,*750*_′ = 0.84; *μ*_*s*,*850*_′ = 0.67), white matter (*μ*_*a*,*750*_ = 0.018; *μ*_*a*,*850*_ = 0.021; *μ*_*s*,*750*_′ = 1.19; *μ*_*s*,*850*_′ = 1.01), and cerebrospinal fluid (*μ*_*a*,*750*_ = 0.004; *μ*_*a*,*850*_ = 0.004; *μ*_*s*,*750*_′ = 0.3; *μ*_*s*,*850*_’ = 0.3)^43^. This light modeling accounts for the wavelength dependence of both the illumination patterns (light fluence), and the collection sensitivity patterns, as published previously with HD-DOT^35^. These modelling steps, combined with superficial signal regression^44,45^ and optimal wavelength choice (λ = 750 and 850 nm) provide accurate unmixing of oxy- and deoxy-hemoglobin^46^.

The atlas-based forward modeling technique eliminates the need for subject-specific forward modeling using individual anatomical images, and results in individual and group level image quality with localization errors on the order of millimeters^47^. Using the atlas anatomy combined with the 188 optode positions, the sensitivity matrix was generated using NIRFAST^48^. The sensitivity matrix was then inverted using Tikhonov regularization^33^. The conversion from differential absorption to differential hemoglobin was made using spectroscopy values from the literature (see Table 1. in reference^33^).

For image reconstruction, the measurement data was converted to voxel space using the inverted sensitivity matrix and spectroscopy parameters described above, which resulted in volumetric time-series data of three hemodynamic contrasts: oxyhemoglobin (∆HbO_2_), deoxyhemoglobin (∆HbR) and total hemoglobin (∆HbT) at a framerate of 1 Hz. All analyses performed on these images utilize the oxyhemoglobin (∆HbO_2_) contrast, unless otherwise specified (see Supplemental Fig. 2 for results with all three hemoglobin contrasts).

**Figure 2:**
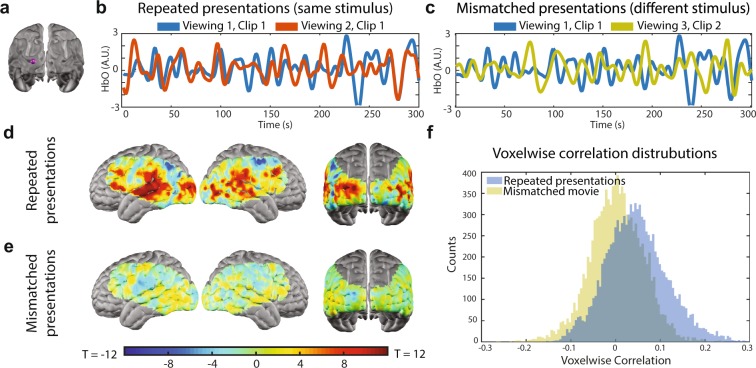
Intra-subject synchronization. (**a**) Seed region (purple sphere) used to extract an exemplar oxy-hemoglobin timeseries during viewing of repeated movie stimuli (**b**) and mismatched movie stimuli (**c**) from a single subject’s data (i.e. two repetitions of the movie stimulus). (**d**) During repeated presentations of the stimulus, the group average of individual viewers’ synchronization maps shows elevated synchronization in regions across the cortical field-of-view, particularly auditory and visual processing regions, as shown in this unthresholded T-map. (**e**) During mismatched stimulus presentations, the synchronization values are greatly reduced, as shown in this unthresholded T-map. (**f**) Voxelwise distributions of Pearson correlation values during repeated and mismatched movie presentations.

Hemoglobin spectroscopy performance was verified using an independent dataset collected from a subset (N = 5) of participants in this study. These participants viewed a rotating wedge consisting of a black and white checkerboard that flickered at a 10 Hz reversal rate to produce an evoked response in visual cortex, following previously published procedures^13,49^. The ratios of the ∆HbO_2,_ ∆HbR_,_ and ∆HbT responses in visual cortex follow previously reported responses obtained with near-infrared tissue spectroscopy using similar stimulation protocols (Supplemental Fig. 2). Utilizing a more traditional stimulation paradigm to evaluate spectroscopy performance confirms that the light modeling and spectroscopy parameters are appropriate for the more novel naturalistic viewing analyses.

### Data analysis of movie responses

The image reconstruction procedure resulted in a volumetric time-series of brain responses time-locked to the stimulus presentation. Two analyses were performed on these HD-DOT images. First, we evaluated the inter- and intra-subject synchronization between multiple viewings of the movie stimulus following procedures previously used with fMRI^20^. Second, we evaluated the correlation between parameterized features of the movie stimulus and the measured cortical responses^19,39^. For correlation maps resulting from both the synchronization and feature-based analyses, the voxelwise correlation coefficients were assessed using the T-statistic. The observed Pearson product-moment correlations were transformed to a normally distributed statistic using the Fisher Z-transformation, $z^{\prime} =0.5[\,\mathrm{ln}(1+r)-\,\mathrm{ln}(1-r)]$. Examples of Z-transformed maps are presented in Supplemental Fig. 1. For a given voxel, the Z-transformed correlation coefficient was then mean subtracted and divided by the standard error, σ/$\sqrt{n}$, where σ is the sample standard deviation, and *n* is the number of images included in a given analysis. The resulting contrast-to-noise T-maps therefore indicate the extent to which an observed correlation coefficient deviates from a null distribution in which there is no observed correlation between signals (e.g. Fig. 2f).

#### Inter- and Intra-Subject Synchronization

To assess the extent to which an individual exhibited synchronized responses across repeated presentations (intra-subject synchronization), as well as the extent to which an individual synchronized with others in the sample (inter-subject synchronization) we performed a correlation analysis. For each voxel, we calculated the correlation coefficient between the voxel’s ∆HbO_2_ timeseries for two separate movie presentations^20^. Repeating this procedure across all voxels in the field-of-view produces a spatial map of synchronization across the cortex.

#### Feature-Based Analysis

The movie stimulus was decomposed into both visual and auditory features in order to more precisely relate features of the stimulus to observed ∆HbO_2_ responses. Visual features included features based on image statistics calculated based on individual movie frames (luminance, flow). Luminance was indexed by the mean pixel intensity for a single frame, after converting the full-color image to a grayscale image^39^. Motion was parametrized by calculating optical flow, using the Lucas-Kanade algorithm for solving the optical flow constraint equation: ${I}_{x}u+\,{I}_{y}v+\,{I}_{t}=0$, where *I*_*x*_, *I*_*y*_, and *I*_*t*_, are spatiotemporal image brightness derivatives, and *u* and *v* are horizontal and vertical optical flow, respectively. The Lucas-Kanade algorithm was implemented using the opticalFlowLK class in the MATLAB Computer Vision Toolbox (noise threshold = 0.0039). For each frame, the average magnitude of optical flow across all pixels was used to track changes in visual motion intensity for the duration of the stimulus.

A second set of visual features included manually coded features: visually presented faces, bodies, and hands. For manually coded features, three human raters viewed the stimulus in 1-second bins and made a binary judgment regarding the presence of the three visual features of interest: Faces, bodies, and hands. Bins during which the raters’ judgments were discordant were subsequently re-evaluated to reach a consensus.

Auditory features included the envelope of the stimulus audio, as well as moment-to-moment changes in the presence of human-generated speech. The envelope of the stimulus audio was used to track overall changes in audio intensity, regardless of the content of the audio, and implemented in MATLAB. Following previously published methods, the envelope of audio intensity was calculated by computing power modulations across 25 frequency bands (center frequencies: 200 Hz − 5 kHz; width: 200 Hz; sampling rate: 50 ms)^36^. Within each band, the logarithm of the power time course was taken, and then all frequency bands were averaged, resulting in a single time course representing the audio envelope of the stimulus audio. The presence of human speech (excluding human-generated non-speech sounds) was manually coded by three raters, using binary judgments on 1-second bins of audio. Discordant judgments were subsequently re-evaluated to reach a consensus.

To model the response to each feature, we convolved the raw feature time-series with a canonical hemodynamic response and then bandpass filtered (0.02 Hz < *f* < 0.5 Hz) the feature time-series to match the measurement filtering parameters^50^. To relate feature dynamics to measured cortical responses, we calculated the temporal correlation between the modeled time-course for each feature and the ∆HbO_2_ response time-course for each voxel across the field-of-view. This procedure generated a spatial map of the correlation strength between the brain responses and each feature time-course. To differentially assess pairs of features, a paired-samples T-test between sets of two feature correlation maps was used to identify cortical regions with differential responses between a pair of features.

## Results

### Inter- and intra-subject synchronization

For a single voxel, intra-subject, between-viewing synchronization is indexed and quantified by the Pearson product-moment correlation coefficient between ∆HbO_2_ timeseries, obtained during two separate repetitions of the same stimulus within the same participant (Fig. 2a–c). Repeating this procedure across the field-of-view generates a correlation map. Averaging these correlation maps across participants (N = 10 participants; 2 stimulus repetitions per participant) reveals regions of elevated correlation coefficients, or synchronization, across the entire HD-DOT field-of-view (Fig. 2d). In particular, elevated correlation coefficients are observed in regions related to auditory and visual processing, underscoring that the strongest correlations observed are due to sensory processing of the multi-modal movie stimulus. For a given participant, intra-subject synchronization was assessed using stimulus repetitions obtained within and across imaging sessions, meaning that an individual participant’s intra-subject synchronization map could include responses measured during movie viewings of the same repeated clip, across multiple days.

Obtaining multiple runs during repetitions of the same stimulus across multiple imaging sessions enables assessment of potential habituation effects, which would result in diminished activation magnitudes as the number of stimulus repetitions increases^51^. In the intra-subject synchronization analysis, these effects would be evident in diminished intra-subject synchronization between viewings across disparate sessions, relative to viewings within a session (i.e. when there have been fewer stimulus repetitions). To assess this potential habituation effect, the intra-subject synchronization analysis was repeated with runs that were (1) obtained within the same session and (2) obtained across separate sessions (Supplemental Fig. 3). While the average value of the Fisher z-transformed correlation was slightly lower across sessions (mean z(r) = 0.038) than it was within sessions (mean z(r) = 0.041), the voxelwise topographies and distributions of correlation coefficients are largely overlapping, (Supplemental Fig. 3), indicating no observed habituation effect across the repeated stimulus presentations in this dataset.

If the observed synchronization between voxelwise responses is related to processing repetitions of the same stimulus, then the magnitude of the correlation should be diminished when the analysis is repeated with ∆HbO_2_ timeseries obtained during disparate viewing conditions (i.e. different movie clips). Indeed, when participants view non-overlapping movie segments, the correlation coefficients are diminished both within a single region (Fig. 2c) and across the entire HD-DOT field-of-view (Fig. 2e). The dramatic reduction in the correlation coefficients is also evident in the distributions of correlation values observed during both matched and mis-matched viewing conditions (Fig. 2f). Voxelwise responses in individual viewers show the greatest reliability, or synchronization, during repetitions of the same stimulus (Fig. 2d).

**Figure 3:**
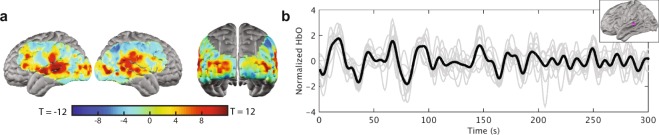
Inter-subject synchronization. (**a**) Pairs of separate viewers also show synchronized cortical responses in regions related to visual and auditory processing during naturalistic viewing, as shown in the unthresholded map of synchronization averaged across all possible pairs of 10 viewers (45 pairs in total). (**b**) Oxy-hemoglobin timeseries from a seed region in the left superior temporal gyrus (inset) for individual viewers (grey lines) and the group average (black line).

Cortical responses measured within single individuals reveal movie-driven responses with high correlation coefficients in regions related to stimulus processing. To assess whether this effect extended beyond individual viewers to disparate pairs of viewers, the synchronization analysis was repeated across all possible pairs of the ten viewers. The voxelwise correlation coefficient between the ∆HbO_2_ time-series in each participant was calculated for each pair of viewers, which revealed a synchronization topography comparable to the intra-subject analysis (Fig. 3). In other words, not only does the naturalistic stimulus reliably drive cortical responses within an individual, it also reliably drives cortical responses across individuals.

### Gaze-Based synchronization modulations

**Figure 4:**
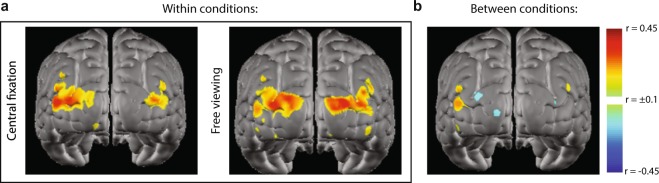
Gaze-based synchronization modulations. A subset of participants (N = 4) viewed an additional repetition of the stimulus under experimenter-imposed viewing conditions, in which participants were instructed to maintain central fixation during movie viewing. Voxelwise maps display raw Pearson correlation coefficients, which provide a quantification of cortical synchronization during both central fixation viewing and free viewing. (**a**) Synchronization is observable when viewing conditions are held constant. (**b**) Synchronization is abolished when comparing across viewing conditions that impose different gaze patterns during naturalistic viewing.

To demonstrate the sensitivity of naturalistic viewing experiments to eye position, a subset of participants (N = 4) in the present work repeated the experiment under modified viewing conditions. These participants viewed the stimulus once under natural conditions, and a second time while maintaining central fixation throughout the entire viewing period, during which a crosshair was overlaid over the center of the movie stimulus. Within both of the viewing conditions, synchronization in visual cortex was preserved, as indexed by the high correlation coefficients in visual cortex (Fig. 4a). However, when the correlation coefficient between ∆HbO_2_ time-series from disparate viewing conditions was calculated, synchronization was diminished, highlighting that the synchronization effect in visual cortex, in part, depends on consistent viewing conditions (Fig. 4b). This result is consistent with the observation that eye position during unconstrained viewing of professionally produced movie is reproducible across viewers^52^.

### Feature-Based analysis

**Figure 5:**
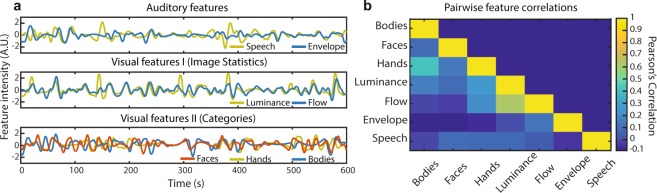
Feature extraction procedure. (**a**) Seven features of varying complexity across visual and auditory processing were extracted from the stimulus using a combination of automated and manual approaches. (**b**) The correlation between pairs of features was generally weak, with the exception of the two visual features based on image statistics: luminance and optical flow.

The feature extraction procedure, applied across visual and auditory modalities, resulted in a set of seven features (Fig. 5). In general, features were not strongly correlated with each other, with the exception of the two features derived from image statistics, luminance and optical flow (Fig. 5b).

**Figure 6:**
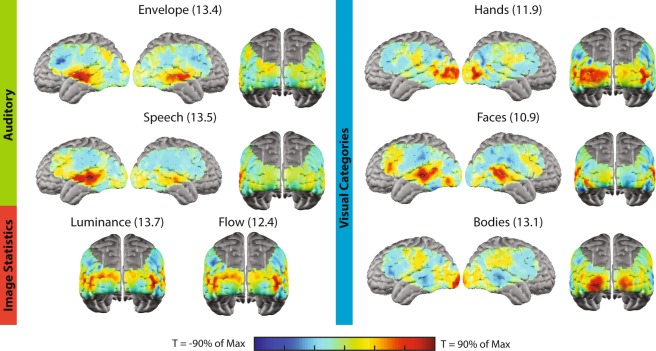
Correlation maps for individual movie features. Maps represent the group averaged map across individual feature maps for each of the 10 subjects included in the analysis. Correlation maps for each of the seven visual and auditory movie features highlight cortical regions related to processing those features during naturalistic viewing. Individual maps are scaled to 90% of the maximum T-statistic for each map, indicated in parentheses next to each map title.

Correlation maps generated from the time-series for each feature category were in agreement with known functional neuroanatomy (Fig. 6)^35,53^, and were evaluated using a T-statistic to identify voxels with correlation coefficients that deviate from a null distribution in which there is no observed correlation between signals (see methods). For instance, in the auditory domain, voxels in the bilateral superior temporal gyrus (STG) had the highest correlation coefficients to the audio envelope feature time-series, while the correlation between voxelwise ∆HbO_2_ and the speech feature revealed a left-lateralized response in the STG and left prefrontal cortex.

For visual features generated by image statistics (luminance and flow), voxels in visual cortex have the highest correlation coefficients between the ∆HbO_2_ time-series in these regions and the feature time-series. Conversely, the set of visual feature time-courses for higher-level visual features revealed patterns of elevated correlation coefficients across broader constellations of regions. For instance, the map of face processing during naturalistic viewing, generated by computing the voxelwise correlation between the ∆HbO_2_ time-series and the visually presented faces time series, not only involved extrastriate visual regions, but also auditory and speech processing regions, underscoring that features were not present in isolation during the naturalistic viewing task^53^. Similarly, the correlation coefficient between voxelwise ∆HbO_2_ and the time-course of visually presented bodies was elevated in voxels in the visual cortex and voxels in the inferior regions surrounding the central sulcus.

### Hierarchical feature contrasts

**Figure 7:**
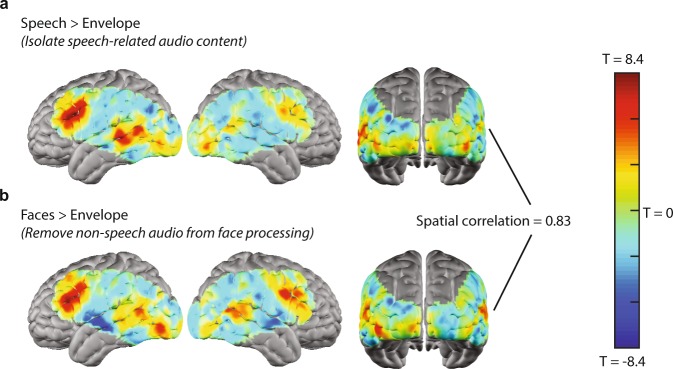
Maps for pairwise feature contrasts. Maps represent the group averaged map across individual feature maps for each of the 10 subjects included in the analysis. Paired T-tests between sets of features aimed at identifying regions related to speech processing during naturalistic viewing reveal that spatially convergent maps can be generated by contrasting disparate, but conceptually related features. (**a**) Contrast between the speech and auditory envelope features. (**b**) Contrast between the visually presented faces and auditory envelope features.

Within the set of features used for functional mapping, individual features differed in complexity. For instance, the audio envelope, a low-level feature, indexed non-specific changes in stimulus audio intensity. Changes in audio intensity during movie viewing may be driven by factors such as environmental sounds, music, or human produced speech. Processing human produced speech is a more complex auditory task with both auditory and linguistic components, and was indexed by a dedicated, higher-level language feature^54^. Consequently, the set of auditory features used in this analysis was both hierarchical and potentially overlapping. To evaluate the relationship between these hierarchical auditory features, a paired T-test was computed between the correlation maps for the audio envelope and speech features (Fig. 7a), resulting in a map of regions that preferentially respond to speech relative to other sounds indexed by the envelope feature. Relative to the correlation map for the speech feature alone (Fig. 6), the contrasted map in Fig. 7 evaluates a region’s selectivity for one feature over another and provided more detailed mapping of regions (e.g. left prefrontal cortex, or Broca’s area) involved in naturalistic speech processing.

Hierarchical features were not limited to features within a single modality. During naturalistic viewing of the stimulus, visually presented human faces co-occurred with auditorily presented human speech. Consequently, the correlation map for the visually presented face feature may serve as a surrogate feature for mapping cortical responses to social information, specifically human speech^55^. However, other ambient sounds may also co-occur with visually presented faces, which are indexed by the auditory envelope. To test this hypothesis, the paired T-test described above was repeated for the face and auditory envelope features (Fig. 7b).

The spatial correlation between the maps generated using these two approaches to isolating speech processing (or contrasted feature maps) was r = 0.83, indicating good agreement. Therefore, by contrasting individual features, cortical responses to movie stimuli were further dissected, evaluating a given region’s preference for one feature over another.

## Discussion

In the present study, we used passive movie viewing, a naturalistic sensory stimulation paradigm, to evaluate the feasibility of measuring synchronized, movie-evoked cortical responses in healthy adult participants using HD-DOT. This synchronization, as indexed by the voxelwise correlation coefficient between oxy-hemoglobin responses measured across repeated viewings, was most prominent in auditory and visual cortex, highlighting that passive movie viewing is an effective tool for engaging distributed, multi-modal cortical regions^20^. Further, the spatial maps of correlation coefficients generated within participants and between participants both demonstrated elevated correlations during repeated stimulus viewings, underscoring that naturalistic stimuli reliably drive cortical activity despite the task’s highly unconstrained conditions. The magnitude of the correlation coefficients was greatly diminished when participants viewed different, non-overlapping movie segments.

While high synchronization was observed both within and between participants over the HD-DOT field of view, this analysis approach did not relate features contained within the movie to specific cortical areas. In order to leverage the reliable cortical responses to the stimulus and relate them to naturalistic information processing, a feature decomposition strategy was employed to parameterize the movie stimulus^19,39,54^. In the initial feature set, seven visual and auditory features of varying complexity were extracted from the stimulus. These features were subsequently used to functionally map cortical regions related to feature-specific processing; highlighting that, despite the richness and concurrent multi-modal stimulation associated with naturalistic tasks, tracking the intensity of individual features encountered during naturalistic viewing provides an effective strategy for parameterizing and mapping the complex movie stimulus.

Both synchronization and feature-based mapping strategies have been successfully incorporated in neuroimaging research using other modalities. Inter- and intra-subject synchronization during naturalistic viewing was first demonstrated using fMRI and has been shown in subsequent studies investigating the reproducibility of movie-evoked cortical responses^20,21^. Similarly, feature-based decomposition of naturalistic stimuli, using both manual and automated decoding approaches, has been incorporated in imaging work in both humans and non-human primates, highlighting that naturalistic tasks are suitable for mapping brain activity in a manner comparable to more constrained stimuli commonly utilized in functional mapping experiments^19,39^.

The present work is extension of these analytic tools to optical neuroimaging modalities, leveraging the relatively high resolution and broad coverage of the superficial cortex that HD-DOT offers compared to sparse fNIRS. Stimuli such as *The Good*, *the Bad*, *and the Ugly*, are narrative movies produced for entertainment; consequently, the “tasks” embedded in processing a feature film are complex, rich, and concurrent. Prior work using fNIRS has also used video stimuli, although generally with the goal of understanding a targeted and constrained information processing task. For example, fNIRS experiments investigating the development of specialized cortical responses to social stimuli have successfully leveraged the richness of video stimuli with human actors^56,57^. Further, these responses have been shown to be sensitive to altered developmental trajectories^58^. Depending on the study, these targeted videos can be optimized for the specific task of interest. On the other hand, because social interactions are inherently rich, multi-modal experiments, video stimuli are an effective tool for recapitulating this richness in a repeatable and controlled manner. By replacing a video stimulus tailored for assessing a specific domain with a feature film, as done in the present work, multiple sensory and cognitive processing domains can be assessed concurrently using a single, integrated movie stimulus. Outside of the laboratory, information is rarely encountered in a single sensory domain under rigidly controlled stimulus presentation parameters, underscoring the ecological relevance associated with free viewing tasks as implemented in this work.

Movie viewing tasks also afford practical advantages for special populations of interest. For instance, toddlers and school age children may find measurements of task-evoked brain activity relying on highly constrained and isolated stimuli to be boring, repetitive, or predictable^17^. Indeed, work using fMRI indicates that naturalistic viewing tasks in toddlers and school age children reduces head motion, a substantial source of artifact^24,25^. Further, the extent to which a child shows synchronized brain responses during naturalistic viewing correlates with behavioral assessments of mathematical and linguistic ability^59^. Future work using naturalistic viewing tasks in conjunction with optical neuroimaging can leverage the practical advantages and scientific value of these paradigms alongside comfortable and wearable instrumentation, such as HD-DOT, that is particularly well suited for pediatric imaging^60^.

One limitation of the naturalistic viewing task, as implemented in this work, is the lack of measured behavioral responses. Behavioral responses can track participant comprehension and attentiveness throughout the task, two variables that have been previously shown to modulate cortical responses measured during naturalistic viewing^27,28,61^. Possible behavioral responses include comprehension assessments following the experiment^27^ and recording eye position during the experiment^62^. Indeed, while eye position during viewing of a professionally produced movie is generally reproducible across subjects, gaze position has been reported to vary in special populations, including participants with Autism spectrum disorder^63^.

In the present work, the importance of eye position during naturalistic viewing was assessed during a separate experiment during which a subset of participants viewed an additional repetition of the stimulus while maintaining central fixation during the entire viewing session. The correlation magnitude between voxelwise responses in visual cortex within viewing conditions indicated synchronized brain responses across participants; however, cortical responses from mismatched viewing conditions did not show the synchronization effect (Fig. 4). In this experiment, participants confirmed their ability to comply with the fixation instructions by self-report. In future work, eye tracking can confirm compliance with experimenter-imposed gaze conditions and provide better characterization of eye position during free viewing.

An additional limitation of the present experiments is the utilization of a single movie clip. Importantly, not all movies are equally suitable for mapping particular features of interest. For instance, an animated movie with animal characters (e.g. *Finding Nemo*) would likely be poorly suited for mapping cortical responses to visually presented hands. Further, a boring or difficult to understand movie (e.g. *Waiting for Godot*) may result in diminished synchronization resulting from poor attentiveness or comprehension^61^. Future work employing naturalistic viewing paradigms can assess the efficacy of differing stimuli in performing functional brain mapping within a given domain of interest, as well as expand the set of features used for a given movie clip. In addition to the low and high-level sensory features used in this work, movie stimuli contain rich social, emotional, and narrative content that engage higher-order brain functions^54,55^. Sex-related differences have been reported in social and emotional processing in imaging and behavioral studies using non-naturalistic designs^64,65^. While the sample in this study was neither sufficiently powered nor balanced to assess potential sex-related effects, naturalistic designs such as passive movie viewing offer a convergent experimental strategy to further explore these sex-related differences in social and emotional processing.

Using optical neuroimaging tools to study the brain during naturalistic viewing conditions has broad applicability for experimental questions demanding rich, engaging stimuli alongside wearable and ergonomic imaging tools^17,66^. Developmental cognitive neuroscience has benefitted from the broad applicability of optical neuroimaging tools for imaging the developing brain^16,56–58,67–69^. Paired with optical neuroimaging, the movie-based imaging paradigm described in this paper provides engaging and ecologically relevant study designs for understanding information processing across the lifespan, highlighting the richness of this paradigm for interrogating “real-life” brain function.

## Supplementary information

Supplemental Figures

## Data Availability

The datasets generated and analyzed during the current study are available from the corresponding author on reasonable request.
