# Supplementary material for: Mapping brain function during naturalistic viewing using high-density diffuse optical tomography
Source: Sci Rep. 2019 Jul 31;9:11115. doi: 10.1038/s41598-019-45555-8 (PMC6668456; doi:10.1038/s41598-019-45555-8)
Supplement: Supplementary file 1 — Supplemental Figures [file 41598_2019_45555_MOESM1_ESM.pdf]

Supplementary Information For

"Mapping brain function during naturalistic viewing using high-density diffuse optical tomography"

Andrew K. Fishell,[a,b] Tracy M. Burns-Yocum,[c] Karla M. Bergonzi[d,e], Adam T. Eggebrecht,[b] Joseph P. Culver[b, f, g]

[a]*Washington University School of Medicine, Division of Biology and Biomedical Sciences, St. Louis, USA*
[b]*Washington University School of Medicine, Mallinckrodt Institute of Radiology, St. Louis, USA*
[c]*Indiana University, Department of Psychological and Brain Sciences, Bloomington, USA*
[d]*University of Pennsylvania, Department of Anesthesia and Critical Care, Philadelphia, USA*
[e]*University of Pennsylvania, Department of Physics, Philadelphia, USA*
[f]*Washington University, Department of Physics, St. Louis, USA*
[g]*Washington University, Department of Biomedical Engineering, St. Louis, USA*

Correspondence:
Joseph P. Culver
Mallinckrodt Institute of Radiology
Washington University School of Medicine
Campus Box 8225
660 S. Euclid Ave
St. Louis, MO 63110
Email: culverj@wustl.edu

Contents

Supplementary Figures

Supplementary Figure 1: Measurement retention 2
Supplementary Figure 2: Inter-subject synchronization with all contrasts 3
Supplementary Figure 3: With and between session synchronization 5

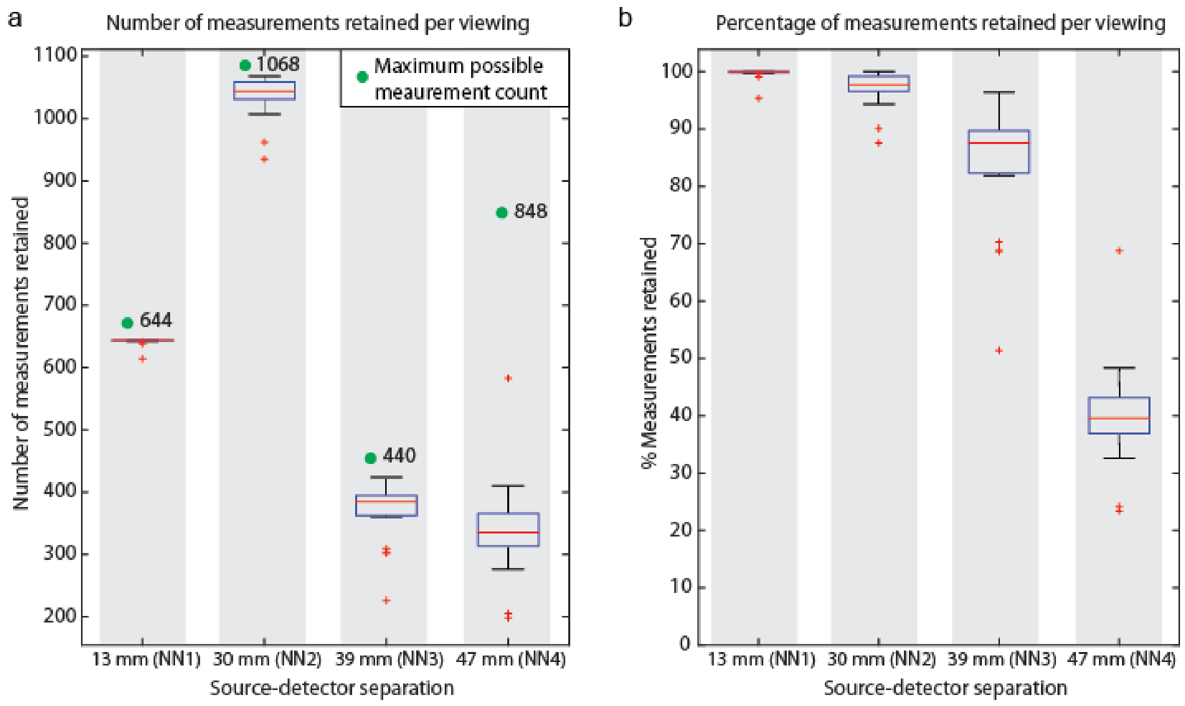

Supplemental Figure 1 Measurement retention across all passive movie viewing runs. **A:** Box plots show the distributions for the number of measurements retained at the first four nearest-neighbor separations, with a mean of 642/644 NN1, 1034/1065 NN2, 368/440 NN3, and 339/848 NN4 measurements retained across all runs. **B:** Box plots show the percentage of measurements retained for the first four nearest-neighbor separations.

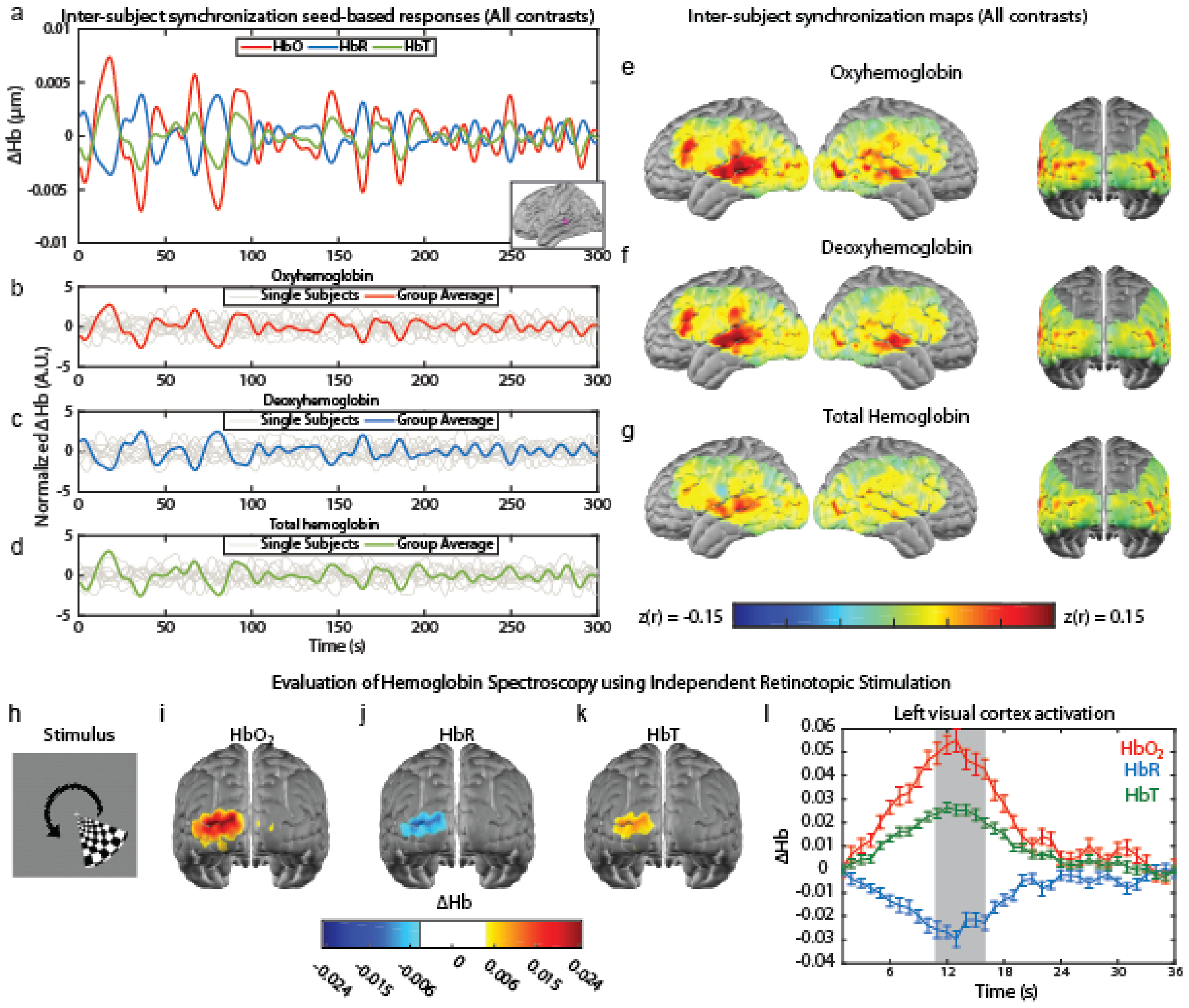

Supplemental Figure 2 Inter-subject synchronization assessed across all oxy-hemoglobin, deoxy-hemoglobin, and total hemoglobin contrasts. **A**: Timeseries for all three contrasts from a seed region in temporal cortex (inset) during a five-minute subset of the passive movie viewing experiment. **B**: The oxy-hemoglobin timeseries from the seed region during passive movie viewing in all 10 individual subjects (grey lines) and the group averaged oxy-hemoglobin response (red line). **C**: The deoxy-hemoglobin timeseries from the seed region during passive movie viewing in all 10 individual subjects (grey lines) and the group averaged deoxy-hemoglobin response (blue line). **D**: The total-hemoglobin timeseries from the seed region during passive movie viewing in all 10 individual subjects (grey lines) and the group averaged total-hemoglobin response (green line). **E**: Group averaged spatial map of correlation coefficients for the inter-subject synchronization analysis performed using the oxy-hemoglobin contrast. Voxel values represent Fisher's Z-transformed correlation coefficients.

F: Group averaged spatial map of correlation coefficients for the inter-subject synchronization analysis performed using the deoxy-hemoglobin contrast. **G**: Group averaged spatial map of correlation coefficients for the inter-subject synchronization analysis performed using the total hemoglobin contrast. **H**: Retinotopic mapping stimulus used for evaluation of hemoglobin spectroscopy in a subset of passive movie viewing participants (N = 5). **I:** Posterior view of the group-level block-averaged oxy-hemoglobin response, averaged over the shaded timepoints in Panel L. **J:** Posterior view of the group-level block-averaged deoxy-hemoglobin response, averaged over the shaded timepoints in Panel L. **I:** Posterior view of the group-level block-averaged total-hemoglobin response, averaged over the shaded timepoints in Panel L. **L:** Activation time traces from left visual cortex for the oxy-hemoglobin, deoxy-hemoglobin, and total hemoglobin contrasts.

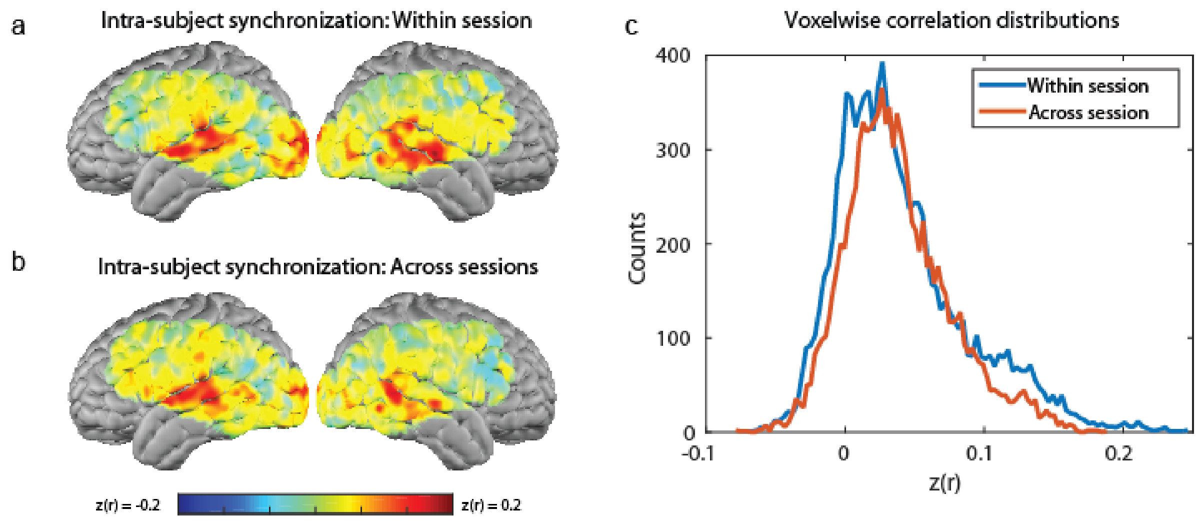

Supplemental Figure 3 Intra-subject synchronization assessed separately for runs collected within the same session and runs collected across separate imaging sessions. **A:** Map of Fisher z-transformed Pearson's correlations for passive movie viewing runs within a single imaging session. **B:** Map of Fisher z-transformed Pearson's correlations for passive movie viewing runs across multiple imaging sessions. **C**: Voxelwise distributions of Fisher z-transformed Pearson's correlations for maps shown in **A** and **B**.
